# Intertumor heterogeneity in 60 pancreatic neuroendocrine tumors associated with multiple endocrine neoplasia type 1

**DOI:** 10.1186/s13023-019-1034-4

**Published:** 2019-02-22

**Authors:** Andreas Selberherr, Oskar Koperek, Philipp Riss, Christian Scheuba, Martin B. Niederle, Reto Kaderli, Aurel Perren, Bruno Niederle

**Affiliations:** 1 Section “Endocrine Surgery”, Division of General Surgery, Department of Surgery, Medical University, Währinger Gürtel 18-20, A-1090 Vienna, Austria; 2 Department of Pathology, Medical University, Währinger Gürtel 18-20, A-1090 Vienna, Austria; 3 Department of Anesthesiology, Medical University, Währinger Gürtel 18-20, A-1090 Vienna, Austria; 4 Institute of Pathology, University of Bern, Murtenstrasse 31, CH-3012 Bern, Switzerland

**Keywords:** MEN-1, Multiple endocrine neoplasia, Pancreatic neuroendocrine tumors, NET, Intertumor heterogeneity

## Abstract

**Background:**

Patients with multiple endocrine neoplasia type 1 (MEN-1) develop multiple pancreatic neuroendocrine neoplasias (PNENs). Size at diagnosis and growth during follow-up are crucial parameters. According to the WHO 2017, grading is another important parameter. The impact of grading compared to size (WHO 2000) on the clinical course needs to be evaluated.

**Methods:**

Sixty PNENs of six patients with MEN-1 were retrospectively evaluated.

**Results:**

Fifty-one tumors with a diameter of < 20 mm were graded as G1. Two of 9 tumors with diameters of ≥20 mm were graded as G2. Tumor size of ≥20 mm correlated significantly with higher proliferation (*p* = 0.000617). Lymph node metastases were documented in two patients with a total of 19 tumors. In one patient, all 13 tumors (diameter: 0.4 to 100 mm) were classified as G1. However, metastases were documented in 9/29 lymph nodes. In the other patient, 5 tumors (3.5 to 20 mm) were classified as G1. The sixth tumor (30 mm) was classified as G2 (Ki-67: 8%). Metastases were revealed in 2/20 lymph nodes.

**Conclusions:**

Tumor size of ≥20 mm seems to correlate with more aggressive MEN-1 related pancreatic disease, regardless of individual proliferation. Tumors ≥20 mm and tumors graded as G2 should be treated surgically regardless of their size.

**Electronic supplementary material:**

The online version of this article (10.1186/s13023-019-1034-4) contains supplementary material, which is available to authorized users.

## Introduction

Patients with multiple endocrine neoplasia type 1 (MEN-1) develop multiple pancreatic neuroendocrine neoplasias (PNENs) which show various sizes and may be functioning (F)- or non-functioning (NF)-PNENs.

Independent of their size mostly F-PNENs are an indication for surgery in an attempt to control hormone excess. The majority of (NF)-PNENs are measuring < 20 mm with a low oncologic risk [1, 2]. The treatment ranges from watchful waiting to partial and total pancreatectomy, the latter resulting in a diabetic metabolic status [1–6]. NF-PNENs ≥20 mm are discussed an indication for surgical intervention [7].

The “20mm size cut-off” was recommended by the WHO 2000 [8] based on clinical follow-up studies of sporadic PNENs showing locally invasive growth as well as local and distant metastasis more often at the time of diagnosis and during clinical follow-up. Size is easily assessed and documented radiologically [9–11], but it seems to be only one of various potential factors that determine the biological tumor behavior. Tumor biology may additionally be characterized by the mitotic count and proliferation index obtained on tissue samples using the WHO grading system [12].

The malignant potential of neuroendocrine tumors (NETs) is divided into three groups (G1, G2 and G3) according to their proliferation rates, measured by mitotic count and by the percentage of cells with immunohistochemically expressed Ki-67 (G1: < 3%; G2: 3–20%, G3: > 20%) [13–15].

In MEN-1 patients, the impact of PNENs grading in correlation with size has not been evaluated to plan either surveillance or surgery; multiple pubmed searches yielded no suggestive results with any combinations of two of the following keywords: MEN-1, multiple endocrine neoplasia, pancreas, grading, size, surgery.

## Materials and methods

Sixty PNENs belonging to 6 MEN-1 patients (2 females, age 38 and 61; 4 males, age 15, 29, 33 and 60) were studied (Table 1).

**Table 1:** Mutation, TNM, surgery, Ki-67 (%) of the largest tumor, and follow-up of 6 MEN-1 patients

Patient	Mutation	Gender	Age	T		N	M	Ki-67%	function	Surgery	Follow-up Status	Years
				ENETS	UICC							
A	Exon 2, del 4 bp (c.247_250delCTGT); - > Termination after amino acid 116	m	29	3	2	0	0	2	F (WDHA-Syndrome)	TP	DF	12
B	Intron 4, G > A -9 bp - > Alternative splicing	f	60	2	2	1	0	8	NF	TP	PD (M?)	10
C	Exon 9, Q405X, CAG > TAG (Gln > Stop) - > Termination after amino acid 404	m	60	2	2	0	0	2	NF	TP	DF	7
D	Exon 3, Codon 179 GAG>AAG (Glu > Lys) - > AS Exchange	m	15	2	1	0	0	3	F (Hyper-insulinism)	DP	DF	29
E	Exon 4, p.I247N, ATT > AAT (Ile > Asn) WORLDWIDE INDEX-CASE	f	38	2	2	0	0	1	NF	TP	DF [+]	4
F	Exon 3, del4bp (amino acid 210/211) - > Termination after amino acid 209	m	33	3	2	1	0	1	F (subclinical Hyper-insulinism)	DP, E	PD (N)	21

*T* tumor classification of the largest tumor, *N* lymph node, *Ki-67* Index in %, *M* distant metastasis, *m* male, *f* female

*F* functioning, *NF* non-functioning

*TP* fotal pancreatectomy, *DP* distal pancreatic resection, *E* enucleation

*DF* disease free, *PD* pogressive disease; [+]: died unrelated to MEN-1

Informed consent was obtained from all individual participants included in the study.

All procedures performed in this study were in accordance with the ethical standards of the institutional review board (approval number: 1053/2013) and with the 1964 Helsinki Declaration and its later amendments.

### Biochemical and clinical pre- and postoperative staging

Preoperatively the biochemical screening and follow-up was performed according to the recently revised ENETS guidelines [16]. In all patients CgA levels were determined preoperatively and during follow-up.

The number, location and appearance of the PNENs were evaluated by endoscopic ultrasound (EUS; fine-needle aspiration cytology was not performed), computerized tomography (CT), and/or by magnetic resonance imaging (MRI) of the pancreas. To exclude distant metastasis somatostatin-receptor (SSR)-mediated scintigraphy was applied at the time of diagnosis.

### Surgery

The indications for surgery were functioning (*n* = 3; organic hyperinsulinism [*n* = 2; patients D and F], Water Diarrhea Hypokalemia Achlorhydria (WDHA) syndrome [*n* = 1; patient A]) or multiple non-functioning tumors > 20 mm (*n* = 3; patients B, C, E).

Total pancreatectomy was performed in patients A, B, C and E (patients B and C suffering from insulin-dependent diabetes mellitus type 2 preoperatively) because of the large amount of PNENs distributed throughout the pancreas without any chance to save “normal” pancreatic tissue. In patient D, a left pancreatic resection was carried out to save parts of the pancreatic body and the head. In patient F, a left pancreatic resection was performed and three PNENs were enucleated from the pancreatic head (Thompson procedure [17]). Extended lymph node dissection was performed in all patients. At the time of surgery, no liver or other distant metastases were documented in any of the patients (cM0). All operations performed were open operations.

### Immunohistochemistry

Each of the 60 PNENs (functioning and non-functioning) and all lymph nodes dissected were evaluated histologically and immunohistochemically.

Staining with chromogranin A (CgA), synaptophysin, Ki-67, Islet-1, TTF1 and CDX2 was performed.

The tumors were classified according to the WHO classification of 2017 [14, 17, 18] and staged according to the European Neuroendocrine Tumor Society (ENETS) consensus proposal of 2006 and the American Joint Committee on Cancer (AJCC)/Union for International Cancer Control (UICC) classification of 2010 [17–19].

Tumor tissue was routinely formalin-fixed and paraffin-embedded. Hematoxylin and eosin (H&E) staining involved 3 μm sections of each block. One representative block of each primary tumor and lymph node metastasis was selected, and 3 μm sections were cut. Immunostainings with chromogranin A (CgA), synaptophysin and against proliferation marker Ki-67 antigen (MIB-1 monoclonal mouse, Novocastra, Newcastle, UK; dilution 1:20), CDX2 (1H9 monoclonal mouse, abcam, Cambridge, UK, undiluted), Islet-1 (1H9 monoclonal mouse, abcam, Cambridge, UK, dilution 1:400) and TTF-1 (SP141 monoclonal rabbit, Ventana, Tucson, Arizona, USA, undiluted) were performed using an automatic immunostainer (Ventana Medical Systems Inc., BenchMark® or BenchMark® ULTRA, Tucson, Arizona, USA). For antigen retrieval, slides for Ki-67, CDX2 and Islet-1 staining were boiled with a commercially available puffer (Ventana Medical Systems Inc., Cell Conditioning 1, Tucson, Arizona, USA) for 256, 256 and 64 min, respectively. In case of Ki-67 staining a commercially available amplification kit (Ventana Medical Systems Inc., Amplification Kit, Tucson, Arizona, USA) was used.

The Ki-67 labeling index with antibody MIB-1 was used for grading and was assessed in 500 tumor cells in areas in which the highest nuclear labeling was observed using an eye grid ocular. The classification was as follows: G1: Ki-67 < 3, G2: Ki-67 3-20, and G3: Ki-67 > 20%.

### Follow-up

All 6 patients were followed clinically and biochemically 4, 7, 10, 12, 21 and 29 years after diagnosis.

Functional imaging by Gallium-DOTANOC-PET-CT was performed in 4 of 6 patients (patients A, B, C and F) 7, 10, 12 and 21 years after surgery. Two patients were clinically cured. However, they refused biochemical and radiological follow-up examinations 4 (patient E) and 29 (patient D) years after pancreatic surgery, therefore cure was not definitively documented.

### Statistical analysis

Comparisons of the distribution of G2 tumors in the group of tumors measuring < 20 mm and ≥ 20 mm and in various subgroups were performed with the chi-square test. Statistical significance was set at a *p*-value of 0.05.

Expression of Islet-1, TTF1 and CDX2 was evaluated semiquantitatively.

## Results

Overall 60 neuroendocrine lesions were documented in the surgical specimens (details can be seen in Table 2 and Additional file 1: Table S1).

**Table 2:** Proliferation index (Ki-67) and immunohistochemical staining of Islet-1, TTF1 and CDX2

Patient	Function	PNEN	Location	Ki-67 (%)	Islet-1 (%)	TTF1 (%)	CDX2 (%)
A	F (WDHA-syndrome)	1	Head	1	+++	+	–
		2	Body	1	+++	–	–
		3	Tail	2	+++	–	–
B	NF	1	Head	2	+++	–	–
		2	Body	8	+++	–	–
		3		1	+	++	–
		4		1	++	–	–
		5		1	+++	–	–
		6	Tail	1	+++	–	–
C	NF	1	Duodenum	1	+	–	++
		2	Head	1	+++	–	–
		3	Body	1	+++	–	++
		4		2	+++	–	–
		5		1	+++	–	–
		6		1	+++	–	–
		7		1	+++	–	–
		8	Tail	1	+++	–	–
		9		1	+++	–	–
		10		1	+++	–	–
		11		1	+++	–	–
		12		1	+++	–	–
		13		1	+++	–	–
		14		1	+++	–	–
		15		1	+++	–	–
D	F (Hyper-insulinism)	1	Body/Tail	1	+++	–	–
		2		1	+++	–	–
		3		3	+++	–	–
		4		2	+++	–	–
		5		1	+++	–	–
E	NF	1	Head	1	n.f.	n.f.	n.f.
		2		1	+++	–	–
		3	Head/Body	1	+++	–	–
		4		1	+++	–	–
		5		1	+++	–	–
		6		1	+++	–	–
		7		1	+++	–	–
		8	Tail	1	+++	–	–
		9		1	+++	–	–
		10		1	+++	–	–
		11		1	+++	–	–
		12		1	+++	–	–
		13		1	+++	–	–
		14		1	+++	–	–
		15		1	+++	–	–
		16		1	+++	–	–
		17		1	+++	–	–
		18		1	+++	–	–
F	F (subclinical Hyper-insulinism)	1	Head/Body	1	+++	–	–
		2		1	+++	–	–
		3		1	+++	–	–
		4		1	+++	–	–
		5		1	+++	–	–
		6		1	+++	–	–
		7		1	+++	–	–
		8		1	+++	–	–
		9		1	+++	–	–
		10		1	+++	–	–
		11		1	+++	–	–
		12		1	+++	–	–
		13	Tail	1	+++	–	–

Positivity of cells (+: ≤ 10%; ++: > 10 to < 100%; +++: 100%)

The tumors were distributed all over the organs: 5 in the pancreatic head, 17 on the junction between the pancreatic head and body, 10 in the body, 5 on the junction between the body and tail, 22 in the tail, and one 5 mm tumor originated in the duodenum.

Imaging by CT or MRI served to localize 10 (16.7%) out of 60 tumors, all measuring ≥10 mm. Five more tumors (5%; between 5 and 9 mm) were documented either by EUS (*n* = 2) or intraoperative ultrasound (IOUS; *n* = 3).

SSR-scintigraphy was negative in all patients (radiologically [r] M0) in terms of distant metastasis.

Forty (66.7%) pancreatic lesions (including the single duodenal lesion) were microadenomas (largest size/diameter: ≤5 mm) [18], 11 (18.3%) were NENs between > 6 mm and < 20 mm, and 9 (15.0%) tumors were ≥ 20 mm, respectively (Table 3).

**Table 3:** Correlation of size and grading - Subgroup analysis

pT		Group		Size (mm)	G1	G2	Ʃ	%
ENETS	AJCC/UICC							
1	1	A	I	≤5	39 + 1^a^	0	40	66.7
			II	6 ≤ 10	8	0	8	13.3
			III	11 < 20	3	0	3	5.0
2	2	B	IV	≥20–40	5	2	7	11.7
3				> 40	2	0	2	3.3
					58	2	60	100

P: pathological; T: tumor, size in mm; G: Grading; G1: Ki-67 < 3; G2: Ki-67 3-20%; ^a^duodenum;

ENETS: European Neuroendocrine Tumor Society

AJCC: American Joint Committee on Cancer; UICC: Union for International Cancer Control

Group A (tumor diameter < 20 mm) vs group B (tumor diameter ≥ 20 mm): *p* = 0.000617

Group I (tumor diameter ≤ 5 mm) vs Groups II to IV (> 6 mm): *p* = 0.041932

Groups I + II (tumor diameter ≤ 10 mm) vs Groups III + IV (> 11 mm): *p* = 0.004018

According to the TNM classification of pancreatic neuroendocrine tumors issued by the ENETS and AJCC/UICC [20], 51 (85.0%) were classified as stage I (pT1, N0, c/rM0), 6 (10%) as IIA (pT2, N0, c/r M0), one as IIB (pT3, N0, M0 c/r), and 2 (3.3%) as IIIB (anyT, N1, c/rM0) according to the ENETS staging system. Applying the AJCC staging system, 51 tumors were staged as IA (pT1, pN0, c/rM0), 7 tumors as IB (pT2, pN0, c/rM0), and the two patients with positive lymph nodes as IIB.

### Size and proliferation (Ki-67) index

Applying the proliferation marker Ki-67, 58 (96.6%) lesions were classified as G1 and two (3%) as G2, respectively. The proliferation rates ranged from 1 to 8%. No tumor was classified as G3.

All 51 tumors with a diameter < 20 mm were graded as G1. Seven of 9 tumors (size = tumor diameter ≥ 20 mm] were graded as G1 and two as G2, respectively.

Table 3 summarizes the various subgroups of tumors based on their different tumor diameters.

Comparing the distribution of G2 tumors in groups I to III (size < 20 mm) and in group IV (≥20 mm), significantly more G2 tumors were found in group IV (0/51 [0%] vs. 2/9 [22%]; chi-square test: *p* = 0.000617).

Comparing groups I + II (size ≤10 mm) and groups III + IV (size > 10 mm), significantly more G2 tumors were found in the group of tumors > 10 mm (0/48 [0%] vs. 2/12 [16.7%]; chi-square test: *p* = 0.004018).

Significance could also be documented comparing group I (size ≤5 mm) with groups II to IV (size > 6 mm; 0/40 [0%] vs. 2/20 [10%]; chi-square test: *p* = 0.041932).

### Individual analyses of 6 patients and follow-up

**Fig. 1:**
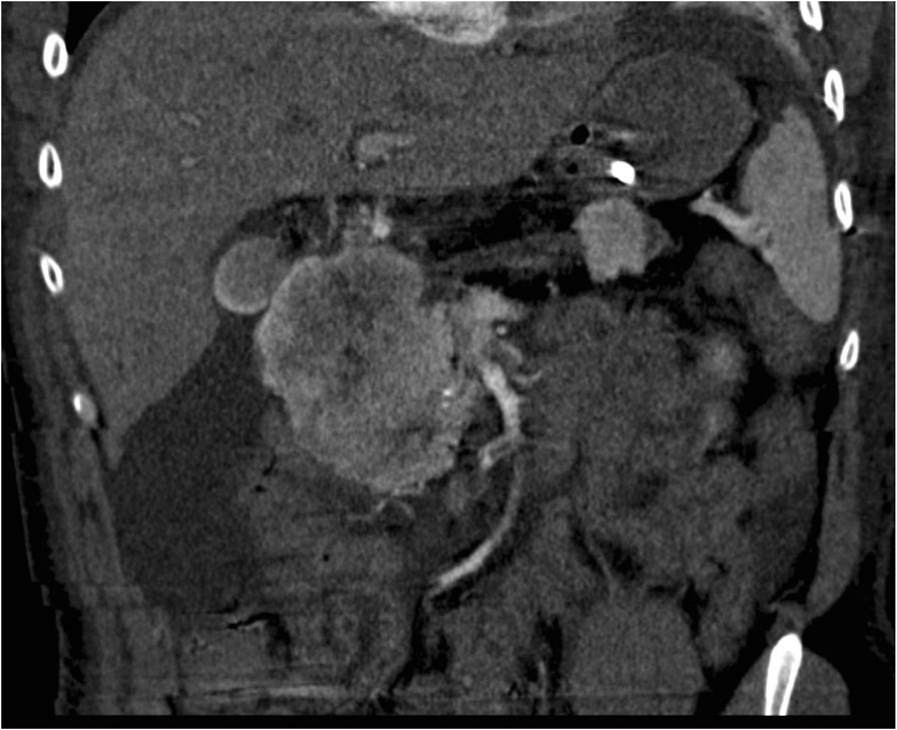
CT-image of two large tumors (80 mm and 40 mm) of patient A that were both immunohistochemically positive for VIP

In **patient A** (3 tumors examined in detail), the largest (80 mm; T3 according to ENETS, T2 according to UICC; G1, N0) and the second largest tumor (40 mm; Fig. 1) were by definition classified as vipomas (proven by VIP expression in more than 70% of the tumor cells; concordant with high blood VIP levels presurgically). Clinically, the patient suffered from severe WDHA syndrome [21].

Twelve years after total pancreatectomy the patient is free of neuroendocrine tumor disease, his insulin-dependent diabetes is well controlled, and he had no complications secondary to it so far.

**Patient B** with 6 PNENs was staged as IIIA. The largest lesion located in the pancreatic head was a moderately proliferating G2 tumor (Ki-67: 8%; TTF1 positiv) 30 mm in diameter (pT2). The proliferation rate of this tumor was identical to the two affected regional lymph nodes (pN1–2/20; Table 4, Ki-67: 6 and 8% in hotspots).

**Table 4:** Patient B: Size, proliferation and immunohistochemistry of the primary tumors and lymph node metastasis

PNEN/ LN	Location	Size (mm)	Ki-67 (%)
1	Head	30	8
2	Body	25	1
3		6.3	1
4		4	1
5		3.5	1
6	Tail	20	1
LN 1			8
LN 2			6

PNEN: Pancreatic neuroendocrine neoplasia; LN = lymph node; Grading: G;

G1: Ki-67 <3%; G2: Ki-67 3-20%)

10 years after total pancreatectomy, the Gallium DOTANOC-PET-CT and MRT revealed multiple small (≤10 mm) liver metastases.

In **patient C**, 15 tumors were examined, including one in the duodenum (which was positive for CDX2). The leading tumor measured 25 mm, corresponding to pT2 N0 cMo. All tumors were graded as G1.

Seven years after total pancreatectomy, the patient is free of neuroendocrine tumor burden.

**Patient D** presented with hypoglycemia. Organic hyperinsulinism was confirmed clinically and biochemically in this patient at age 15. Preoperatively, one tumor was diagnosed in the pancreatic tail (20 mm), while three others (15, 6, 5 mm) were localized by intraoperative sonography in the pancreatic body. After left pancreatic resection, five neuroendocrine neoplasias were described in final histology. The largest (20 mm) with 3% positivity for Ki-67 presented by definition as a low G2 NET. More than 70% of the neuroendocrine cells of this tumor showed immunopositivity for insulin (=insulinoma).

Twenty-nine years after surgery, the patient is clinically cured. Now 42 years of age, he refuses any follow-up examinations.

Seventeen tumors (all smaller than 10 mm) were evaluated in the specimen obtained from **patient E** after total pancreatectomy. None showed a proliferation rate higher than 1%, corresponding to G1. The original histopathological report described another non-functioning tumor of 22 mm in diameter (the only one located by CT preoperatively) in the pancreatic head with a Ki-67 of < 1%. Therefore, the tumor was classified as pT2.

Clinically free of symptoms, she died of liver cirrhosis based on alcohol abuse four years later.

**Patient F** had 13 tumors ranging from less than 1 mm in size to 100mm (!). The large tumor described in CT was located in the pancreatic tail. All lesions, including the large one, were graded as G1. At the time of surgery, lymph node metastases were diagnosed in 9 of 29 lymph nodes. All lymph nodes were invaded by a G1 tumor. The patient had (subclinical) hyperinsulinism. Immunohistochemically, the largest tumor was negative for insulin, but some smaller tumors showed insulin-positive neuroendocrine cells dispersed (less than 70%) in the lesions.

Twenty-one years after subtotal pancreatic left resection and enucleation of three pancreatic head tumors and lymph node dissection, low normal fasting glucose levels correspond to high normal insulin and C-peptide levels. The patient is free of clinical symptoms. Ga-DOTANOC-PET-CT reveals multiple lymph node metastases in the upper abdomen.

## Discussion

All NENs are potentially malignant lesions [22]. The majority of PNENs are histologically well differentiated and slow-growing tumors that differ in their biological behavior. In the WHO 2000 classification, NF- and F-tumors (except insulinoma) were thought to show low-grade malignancy [23]. Clinical behavior is influenced by various clinical-pathological features such as size, local−/angioinvasion and histological differentiation [24].

Currently, the ENETS and the WHO 2017 each propose a formal classification for PNENs based on proliferative tumor activity as measured by mitotic count and the expression of nuclear antigen Ki-67, subdividing the NENs into G1, G2 and G3, respectively [14, 18]. Grading is combined with site-specific (TNM) staging to improve prognostic strength.

To our knowledge, the prognostic impact of PNEN grading in correlation to size (pT) has not yet been evaluated in MEN-1 because the number of patients with pancreatic surgery is low. However, as shown here there seem to be no significant differences in the biological behavior of sporadic and hereditary PNENs.

Due to the genetic background of MEN-1, every single neuroendocrine cell of the pancreas is a potential progenitor of a NEN. Therefore, organs are pervaded by neuroendocrine micro- and macro-lesions in up to 90% of genetically affected patients [19]. As expected, the majority of neuroendocrine lesions in the six MEN-1 patients were non-functioning and developed predominantly as microadenomas (≤ 5 mm in diameter; 66.7%).

Fifty-eight (96.6%) of the 60 lesions were graded as G1 and two (3.3%) tumors as G2 [14, 18]. No lesion was graded as G3. G2 tumors were found only in lesions ≥20 mm (ENETS/AJCC pT2), while all tumors < 20 mm (ENETS/AJCC pT1) were graded as G1.

Within one pancreatic gland, NENs of various sizes and different Ki-67 indices were found, demonstrating intertumor heterogeneity within one patient. These findings underline the observation that size – an important parameter for the definition of T in the TNM classification – appears to be an independent predictor of survival, and the evaluation of Ki-67 alone cannot be utilized for this purpose. This is comparable to the situation in sporadic PNENs [25]. Grading may help to better estimate metastasizing capacity. As every single tumor is a potential risk for systemic disease [26], early diagnosis and surgical excision of MEN-1-related PNENs can improve survival [3].

Imaging with novel radiolabeled somatostatin analogs (Ga^68^-DOTANOC), PET/CT or MRI allows to measure size, to verify local invasion of the primary tumor(s), and to evaluate the presence of metastatic disease. Functioning imaging is the key element in the management of patients with MEN-1 to determine appropriate therapeutic strategies. With regard to our series, somatostatin receptor imaging was performed in all patients and yielded negative results in respect to distant metastases before surgery. During follow up DOPA-Peptide-PET-CTs were used in all patients and could detect metastatic lesions in two, which amended the treatment regimens.

The indication, timing and extent of surgery in NF-PENs has to be individualized on the basis of size and proliferation activity, keeping in mind the potential morbidity of pancreatic resection and the risk of long-term insulin dependence.

Tumor size is easily assessed with EUS or cross-sectional imaging, while Ki-67 grading on histological samples obtained by EUS-guided fine-needle aspiration is technically complex [27].

The current analysis is in accordance with some authors who currently agree that NF-PNENs ≤10 mm can be followed conservatively: all 48 NF-NENs analyzed in this investigation were graded as G1. The management of NF-PNENs sized 11 to < 20 mm is a matter of debate. The progression-free survival may be identical in patients undergoing active surveillance compared to surgery [28, 29].

The procedures currently discussed are either resection or follow-up. Lopez et al. have recommended surgical treatment for NF-PNENs in MEN-1 with a size between 10 and 20 mm, should rapid progression – defined by 5 mm tumor growth annually – be observed [30]. The current findings indirectly emphasize the recommendation that NF-PNENs ≥20 mm (pT2) should be treated surgically as they likely yield a more aggressive clinical course, especially if an elevated Ki-67 index is documented additionally.

## Conclusion

This small-scale series with 60 NENs harvested from 6 MEN-1 pancreatic glands adds additional information regarding the importance of size (pT) in combination with proliferation (G). In this series, significantly more tumors ≥20 mm were classified as G2 (Ki-67 index: > 2 to 20%) yielding indirectly a higher capacity of malignancy.

When tumors reach the cutoff (20 mm; leading tumors), it may be recommended to obtain tissue specimens of this tumor to select patients at risk of highly proliferating neoplasms who may require early surgical intervention to prevent invasive growth, regional and distant metastasis. PNENs with Ki-67 positive cells > 2% should probably be treated surgically early on, regardless of their size.

## Additional file

Additional file 1: **Table S1.** Pancreatic neuroendocrine neoplasia in MEN-1: Size, TNM, proliferation. A, B, C, E: total pancreatectomy; D: left pancreatic resection; F: Thompson procedure. (DOCX 22 kb)

## Abbreviations

AJCC: American Joint Committee on Cancer
CDX 2: Caudal related homeobox transcription factor 2
CgA: Chromogranin A
CT: Computerized tomography
DOTANOC: DOTA-Na13-Octreotide
ENETS: European neuroendocrine tumor society
EUS: Endoscopic ultrasound
Ga^68^: Positron-emitting Gallium isotope (half-life: 68 min)
IOUS: Intraoperative ultrasound
Ki-67: Kiel – 67
MEN: Multiple endocrine neoplasia
MIB-1: Molecular immunology borstel – 1
MRI: Magnetic resonance imaging
NEN: Neuroendocrine neoplasia
NET: Neuroendocrine tumor
PET: Positron emission tomography
PNEN: Pancreatic neuroendocrine neoplasia
PNET: Pancreatic neuroendocrine tumor
SSR: Somatostatin receptor
TNM: Tumor (T), lymph node (N), metastasis (M) – classification
TTF 1: Thyroid transcription factor 1
UICC: Union for International Cancer Control
VIP: Vasoactive intestinal peptide
WDHA: Water Diarrhea Hypokalemia Achlorhydria
WHO: World Health Organization
