# Supplementary material for: Intertumor heterogeneity in 60 pancreatic neuroendocrine tumors associated with multiple endocrine neoplasia type 1
Source: Orphanet J Rare Dis. 2019 Feb 22;14:54. doi: 10.1186/s13023-019-1034-4 (PMC6387504; doi:10.1186/s13023-019-1034-4)
Supplement: Supplementary file 1 — Table S1. Pancreatic neuroendocrine neoplasia in MEN-1: Size, TNM, proliferation. A, B, C, E: total pancreatectomy; D: left pancreatic resection; F: Thompson procedure. (DOCX 22 kb) [file 13023_2019_1034_MOESM1_ESM.docx]

Table 2a. Pancreatic neuroendocrine neoplasia in MEN-1: Size, TNM, proliferation. A, B, C, E: total pancreatectomy;

D: left pancreatic resection; F: Thompson procedure.

| Patient | Function | PNEN | Location | Size (mm) | T | | N | M | Ki 67  (%) | G  (WHO 2017) |
| --- | --- | --- | --- | --- | --- | --- | --- | --- | --- | --- |
|  |  |  |  |  | E | U |  |  |  |  |
| A | F  (WDHA-syndrome) | 1 | Head | 80 | 3 | 2 | 0 | 0 | 1 | 1 |
|  |  | 2 | Body | 2 |  |  |  |  | 1 | 1 |
|  |  | 3 | Tail | 40 |  |  |  |  | 2 | 1 |
| B | NF | 1 | Head | 30 | 2 | 2 | 1 | 0 | 8 | 2 |
|  |  | 2 | Body | 25 |  |  |  |  | 1 | 1 |
|  |  | 3 |  | 6.3 |  |  |  |  | 1 | 1 |
|  |  | 4 |  | 4 |  |  |  |  | 1 | 1 |
|  |  | 5 |  | 3.5 |  |  |  |  | 1 | 1 |
|  |  | 6 | Tail | 20 |  |  |  |  | 1 | 1 |
| C | NF | 1 | Duodenum | 5 | 2 | 2 | 0 | 0 | 1 | 1 |
|  |  | 2 | Head | 5 |  |  |  |  | 1 | 1 |
|  |  | 3 | Body | 25 |  |  |  |  | 2 | 1 |
|  |  | 4 |  | 6 |  |  |  |  | 1 | 1 |
|  |  | 5 |  | 2.1 |  |  |  |  | 1 | 1 |
|  |  | 6 |  | 2 |  |  |  |  | 1 | 1 |
|  |  | 7 |  | 1.3 |  |  |  |  | 1 | 1 |
|  |  | 8 | Tail | 4 |  |  |  |  | 1 | 1 |
|  |  | 9 |  | 4 |  |  |  |  | 1 | 1 |
|  |  | 10 |  | 3.5 |  |  |  |  | 1 | 1 |
|  |  | 11 |  | 3.5 |  |  |  |  | 1 | 1 |
|  |  | 12 |  | 2.8 |  |  |  |  | 1 | 1 |
|  |  | 13 |  | 2.7 |  |  |  |  | 1 | 1 |
|  |  | 14 |  | 2.6 |  |  |  |  | 1 | 1 |
|  |  | 15 |  | 2.4 |  |  |  |  | 1 | 1 |
| D | F  (Hyper-insulinism) | 1 | Body/Tail | 6.4 | 2 | 1 | 0 | 0 | 1 | 1 |
|  |  | 2 |  | 20 |  |  |  |  | 3 | 2 |
|  |  | 3 |  | 15 |  |  |  |  | 2 | 1 |
|  |  | 4 |  | 1.1 |  |  |  |  | 1 | 1 |
|  |  | 5 |  | 4.7 |  |  |  |  | 1 | 1 |
| E | NF | 1 | Head | 22 | 2 | 2 | 0 | 0 | 1 | 1 |
|  |  | 2 |  | 4 |  |  |  |  | 1 | 1 |
|  |  | 3 | Head/Body | 8.8 |  |  |  |  | 1 | 1 |
|  |  | 4 |  | 4.8 |  |  |  |  | 1 | 1 |
|  |  | 5 |  | 1.1 |  |  |  |  | 1 | 1 |
|  |  | 6 |  | 6.1 |  |  |  |  | 1 | 1 |
|  |  | 7 |  | 3.9 |  |  |  |  | 1 | 1 |
|  |  | 8 | Tail | 1 |  |  |  |  | 1 | 1 |
|  |  | 9 |  | 1 |  |  |  |  | 1 | 1 |
|  |  | 10 |  | 2.5 |  |  |  |  | 1 | 1 |
|  |  | 11 |  | 2.9 |  |  |  |  | 1 | 1 |
|  |  | 12 |  | 1.3 |  |  |  |  | 1 | 1 |
|  |  | 13 |  | 6.2 |  |  |  |  | 1 | 1 |
|  |  | 14 |  | 2.1 |  |  |  |  | 1 | 1 |
|  |  | 15 |  | 1.3 |  |  |  |  | 1 | 1 |
|  |  | 16 |  | 1.2 |  |  |  |  | 1 | 1 |
|  |  | 17 |  | 1.7 |  |  |  |  | 1 | 1 |
|  |  | 18 |  | 2.1 |  |  |  |  | 1 | 1 |
| F | F  (subclinicalHyper-insulinism) | 1 | Head/Body | 2.8 | 3 | 2 | 1 | 0 | 1 | 1 |
|  |  | 2 |  | 12.3 |  |  |  |  | 1 | 1 |
|  |  | 3 |  | 3.2 |  |  |  |  | 1 | 1 |
|  |  | 4 |  | 13.6 |  |  |  |  | 1 | 1 |
|  |  | 5 |  | 4.6 |  |  |  |  | 1 | 1 |
|  |  | 6 |  | 2.1 |  |  |  |  | 1 | 1 |
|  |  | 7 |  | 8 |  |  |  |  | 1 | 1 |
|  |  | 8 |  | 6.9 |  |  |  |  | 1 | 1 |
|  |  | 9 |  | 0.4 |  |  |  |  | 1 | 1 |
|  |  | 10 |  | 0.8 |  |  |  |  | 1 | 1 |
|  |  | 11 |  | 1.4 |  |  |  |  | 1 | 1 |
|  |  | 12 |  | 0.7 |  |  |  |  | 1 | 1 |
|  |  | 13 | Tail | 100 |  |  |  |  | 1 | 1 |

F: functioning; NF: non-functioning. E: ENETS; U: UICC. G1: Ki-67 <3%; G2: Ki-67 3-20%
